# Inhibition of PDE2 reverses beta amyloid induced memory impairment through regulation of PKA/PKG-dependent neuro-inflammatory and apoptotic pathways

**DOI:** 10.1038/s41598-017-08070-2

**Published:** 2017-09-21

**Authors:** Li Wang, Yilixiati Xiaokaiti, Gang Wang, Xiaoxiao Xu, Ling Chen, Xianfeng Huang, Li Liu, Jianchun Pan, Shuqun Hu, Zhuoyou Chen, Ying Xu

**Affiliations:** 1 Clinical Laboratory, Xuzhou No. 1 People’s Hospital, Xuzhou, Jiangsu Province 221002 China; 2 Department of Pharmaceutical Sciences, School of Pharmacy and Pharmaceutical Sciences, State University of New York at Buffalo, Buffalo, NY 14214 USA; 3 Department of Clinical Pharmacy, Hangzhou First People’s Hospital, Nanjing Medical University, Hangzhou, Zhejiang Province 310006 China; 4 Brain Institute, School of Pharmacy, Wenzhou Medical University, Wenzhou, Zhejiang Province 325021 China; 5 School of Pharmaceutical Engineering & Life Sciences, Changzhou University, Changzhou, Jiangsu Province 213164 China; 6 Xuzhou Medical University, Xuzhou, Jiangsu Province 221006 China; 7 Changzhou No. 2 People’s Hospital, the Affiliated Hospital of Nanjing Medical University, Changzhou, Jiangsu Province 213161 China

## Abstract

Beta amyloid peptides (Aβ) are known risk factors involved in cognitive impairment, neuroinflammatory and apoptotic processes in Alzheimer’s disease (AD). Phosphodiesterase 2 (PDE2) inhibitors increase the intracellular cAMP and/or cGMP activities, which may ameliorate cognitive deficits associated with AD. However, it remains unclear whether PDE2 mediated neuroapoptotic and neuroinflammatory events, as well as cognitive performance in AD are related to cAMP/cGMP-dependent pathways. The present study investigated how the selective PDE2 inhibitor BAY60-7550 (BAY) affected Aβ-induced learning and memory impairment in two classic rodent models. IL-22 and IL-17, Bax and Bcl-2, PKA/PKG and the brain derived neurotropic factor (BDNF) levels in hippocampus and cortex were detected with immunoblotting assay. The results showed that BAY reversed Aβ-induced cognitive impairment as shown in the water maze test and step-down test. Moreover, BAY treatment reversed the Aβ-induced changes in IL-22 and IL-17 and the ratio of Bax/Bcl-2. Changes in cAMP/cGMP levels, PKA/PKG and BDNF expression were also prevented by BAY. These effects of BAY on memory performance and related neurochemical changes were partially blocked by the PKG inhibitor KT 5823. These findings indicated that the protective effects of BAY against Aβ-induced memory deficits might involve the regulation of neuroinflammation and neuronal apoptotic events.

## Introduction

Alzheimer’s disease (AD) is often characterized by accumulation of beta amyloid peptides (Aβ) and neurofibrillary tangles (NFTs) in the brain, widespread cortical neuronal loss and the progressive memory impairment^1^. The accumulation of Aβ, particularly Aβ 1-42, and their deposition in insoluble plaques are the major neuropathological hallmarks of AD^2^. Aβ is believed to induce inflammatory reaction, trigger neuronal apoptosis, inhibit cortical and hippocampal remodeling, and therefore result in memory impairment^3^. Inhibition of hippocampal Aβ 1-42 is necessary, but not sufficient for preventing memory impairment in the early stage of AD. The specific mechanisms that lead to AD and memory deficits remain unclear, which result in lack of currently effective treatments of AD. Therefore, there has been on-going research to identify novel targets for further development of treatment strategies against AD.

Cyclic nucleotide (namely cAMP and cGMP) concentrations are tightly controlled by phosphodiesterases (PDEs)^4^. One member of particular interest within the central nervous system is PDE2 (also known as PDE2A), primarily due to its high expression in the limbic nervous system, areas associated with memory performance and cognitive functions^5,6^. PDE2A is a dual-substrate PDE, but preferentially targets cGMP in the presence of high cAMP levels. A low concentration of cGMP inhibits PDE2A and thereby increases a local pool of cAMP, whereas higher concentration of cGMP activates it, allowing a cGMP-mediated decrease in cAMP signaling^7^. Cyclic GMP can activate PDE2 by binding to the regulatory domain of PDE2, thereby increasing its rate of hydrolysis of both cGMP and cAMP. Cyclic GMP in a low concentration inhibits PDE2A activity, once accumulates to a high concentration, cGMP activates PDE2A thereby decrease both of cAMP and cGMP level. The second messenger cAMP, synthesized from ATP by adenylate cylcase (AC), on its own can activate protein kinase A (PKA) which phosphorylates the cAMP response element-binding protein (CREB), and can thereby affect the transcription of genes related to synaptic plasticity and survival, like brain-derived neurotrophic factor (BDNF)^8^.

Inflammation has been observed in neurodegenerative disorders, such as AD. Accumulation of Aβ has been shown to cause inflammation, leading to the activation of microglia around Aβ plaques. These activated microglia likely contribute to the increased levels of cytokines and chemokines, including interleukin-1 (IL-1), IL-6, and tumor necrosis factor α (TNF-α), as they were observed in AD brains^9,10^. Inflammation factors are the main reason which causes neuron cell apoptosis in AD. Several innate immune molecules can contribute to cytotoxic and cytolytic activities and must be controlled to avoid neuronal loss and excessive inflammation. There is significant infiltration of IFNγ- and IL-17-producing T cells and NKT cells in older APP/PS1 mice after 14 days of respiratory infection. This is accompanied by increased glial activation and amyloid-β deposition^11^. Neuro-inflammation factors lead to apoptotic neuron cell death. *In vivo* and *in vitro* studies have demonstrated that the activation of microglia cell triggered by the binding of Aβ and TLR-4, promotes the aberrant release of inflammatory mediators includinginterleukin-1β (IL-1β), tumor necrosis factor-α (TNF-α) and reactive oxygen species (ROS) etc.^12^. This mediator scanning results in neuronal degeneration and accelerates pathological progression of AD^13^.

The mechanisms underlying Aβ-induced memory disorders involving neuroinflammation and neuronal apoptosis are still poorly understood. The present study provided some reliable evidences for the PDE2-mediated effect on Aβ-induced memory disorders in the preclinical AD study.

## Materials and Methods

### Animals

Male ICR mice (8 weeks) weighting 22–25 g were used (Harlan, Indianapolis, IN) for the experiments. Mice were kept in a temperature-controlled room under standard laboratory conditions, with a normal 12 h light/12 h dark cycle. All animals were allowed at least 1-week for habituation before any treatments. Water and food were freely available in their home cages. All the experiments were carried out from 8:30 am to 4:30 pm in a quiet room according to the “NIH Guide for the Care and Use of Laboratory Animals” (NIH Publications No. 80–23, revised 1996) and were approved by the Institutional Animal Care and Use Committee of New York State University at Buffalo (USA).

### Surgery for brain cannula implantation

Animals were anaesthetized with ketamine and xylazine (100 and 10 mg/kg i.p., respectively) and placed in a stereotaxic frame with flat-skull position. The stainless steel guide cannulas were implanted bilaterally into the CA1 area of hippocampus (anterior-posterior −1.7 mm from bregma, mediolateral ± 0.8 mm from midline and dorsoventral −2.0 mm from dura). The cannulas were anchored to the skull with dental cement and then stainless steel styles were inserted into the guide cannulas to maintain patency for microinjection. Mice were given bilateral microinjections of 2 μl Aβ 1-42 (0.4 μg in 1 μl/side), corresponding volumes were infused bilaterally at the rate of 0.25 μl/min using a syringe pump^14^. The mice were then allowed to recover for 4 days and were handled every other day to reduce stress associated with handling at the time of testing.

### Chemicals and drug administration

**Figure 1:**
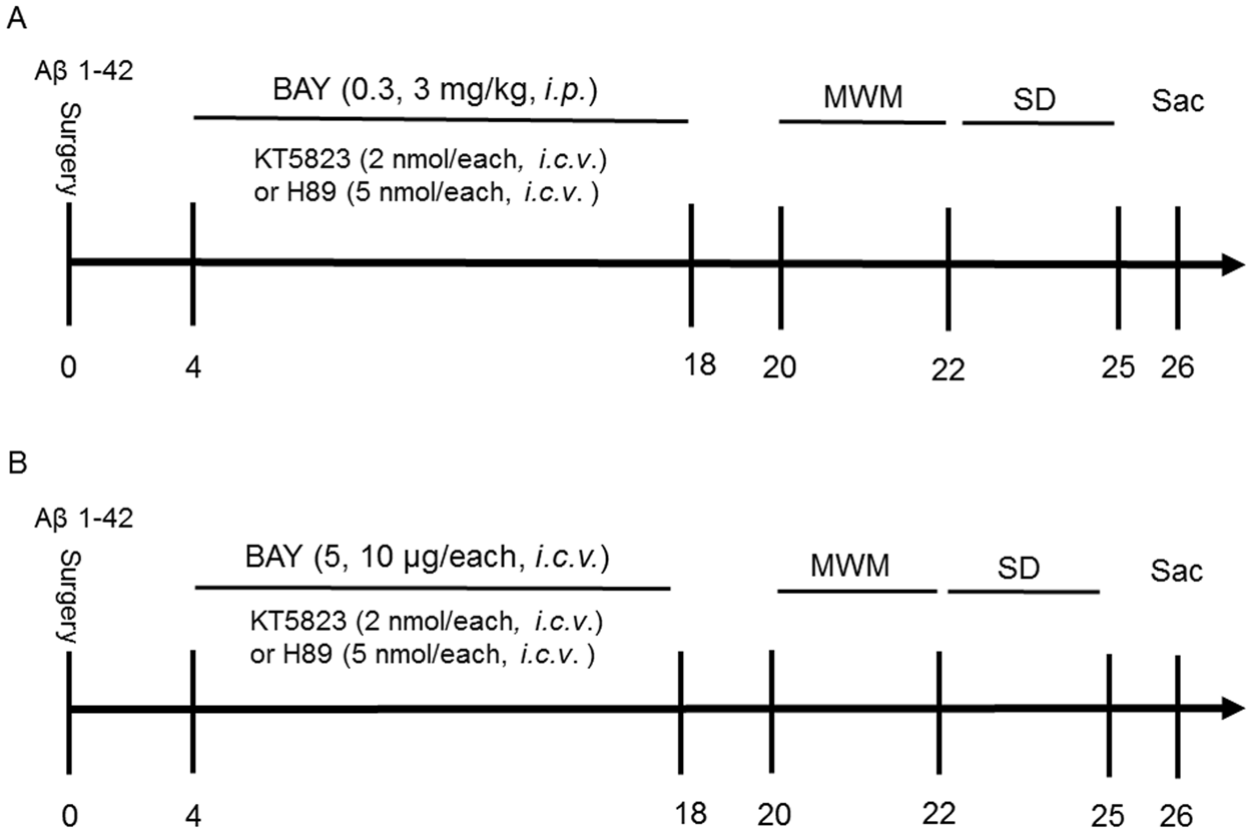
Experimental timeline for drug treatments. Aβ 1-42 or vehicle was microinfused into cerebroventricle (0.4 μg, 1 μl, *i.c.v*.) 4 days after cannule implantation. BAY 60-7550 (0.3 and 3 mg/kg via *i.p*. (**A**); 5 and 10 μg, 2 μl via *i.c.v*. (**B**)) or vehicle was given once per day for 14 days. KT5823 (1 μl, 2 nmol), and H89 (1 μl, 5 nmol) were administered 30 minutes before treatment with BAY 60-7550. All the behavioral tests were performed 24 hours after last drug treatment. Behavioral tests were carried out on days 20–22, then the mice were sacrificed and biochemical assays were performed. MWM, Morris water maze; Sac, sacrifice; SD, Step-down passive avoidance test. N = 8–10 for each experiment.

Aβ 1-42 (Aβ, rPeptide, USA) was dissolved in 0.9% sterile saline, at a final concentration of 0.4 mg/ml (0.4 μg, 1 μl, microinfusion into cerebroventricle, *i.c.v*). The Aβ 1-42 solution was incubated at 37 °C for 4 days to obtain aggregated Aβ before use^15,16^. BAY60-7550 (BAY) and KT5823 were obtained from Cayman Chemical (Ann Arbor, MI, USA). H89 were purchased from Calbiochem (San Diego, CA, USA). BAY was dissolved in 0.5% dimethyl sulfoxide and was administered via the intraperitoneal route (*i.p*.) or via microinjection into cerebroventricle (*i.c.v*). BAY (0.3 and 3 mg/kg for *i.p*. 0.2 mL/20 g (body weight), 5 and 10 μg for *i.c.v*, 1 μL/side) or vehicle was given once per day for 14 days. KT5823 (1 μl, 2 nmol), and H89 (1 μl, 5 nmol) were administered 30 minutes before treatment with BAY. Subject number is 10 mice/group for each condition. All the behavioral tests started 24 hours after last drug treatment (Fig. 1).

The primary antibodies of anti-PKA, anti-PKG, anti-IL-22, anti-IL-17, anti-Bcl-2 and anti-Bax were purchased from Abcam Biotechnology Company (Abcam, Cambridge, MA). The anti-BDNF and anti-β-actin and the related secondary antibodies (anti-mouse lgG or anti-rabbit lgG) were purchased from Santa Cruz Biotechnology Incorporated (Santa Cruz, CA). The cGMP and cAMP ELISA kits were purchased form ENZO life science Company (New York, NY, USA).

### Behavioral procedures

The Morris water maze test was carried out as described previously^17^. The apparatus consisted of a circular, plastic pool (95 cm diameter × 25 cm high) located in a well-illuminated room with external cues visible from the inside of the pool, which was filled with opaque water (26 ± 1 °C). A hidden circular platform (8.5 cm diameter × 15.5 cm high) was submerged 1 cm under the water in one of four quadrants. The acquisition trials (training to escape to the hidden platform) were carried out for six blocks including 3 trials separated by 20 min inter-block intervals during which the platform was visible in the same location relative to the distal cues in the room, six learning blocks were given before probe trial (Fig. 1A,B). The probe trial was performed with the platform removed to measure spatial memory. The number of entries into, the percentage (%) of time spend in the target quadrant and latency to the target quadrant where the platform was previously located were determined 1 h and 24 h after training.

The step-down passive avoidance test was carried out in mice using a chamber containing a wooden platform on one side of the grid floor^18^, electric shocks were delivered to the grid using an isolated pulse stimulator. During the training, mice were individually placed on the platform and subjected to a foot shock (0.4–0.8 mA, 40 V, 0.5 s, 50 Hz, 20 sec intertribal interval) when they completely descended to the grid floor. This procedure was repeated immediately and again 1 h after the initial training. Mice that stayed on the platform for over 60 s were considered to have learned the task and were removed to their home cages, without being given further shocks. Retention tests were carried out 24 h after the last training session. For all retention tests, each mouse was placed on the platform and the step-down latency was recorded, with an upper cut-off time of 300 s.

### cAMP and cGMP measurement in hippocampus and prefrontal cortex

The cGMP (cyclic GMP Complete Enzyme Immunoassay Kit, ADI-901–164, ENZO life science) and cAMP (cyclic AMP Complete Enzyme Immunoassay Kit, ADI-901-163, ENZO life science) ELISA kits were used for measuring cAMP and cGMP levels in the hippocampus and prefrontal cortex. Briefly, mice were sacrificed by CO_2_ narcosis and were decapitated. The brains were quickly removed, and the hippocampus, prefrontal cortex were carefully micro-dissected on ice using a coronal brain slicer matrix and coordinates from a mouse atlas as a reference. Dissected tissue samples were collected in labelled, pre-weighed, 1.4 ml U-bottom polycarbonate tubes (Micronic, Aston, PA; Cat. no. MP22502). 50 μM of the conjugate and antibody solution was added to 100 μL of tissue-derived supernatant in a 96-well plate. The mixture was allowed for shaking incubation (500 rpm) at room temperature for 120 min. After washing out three times, pNpp substrate solution (200 μL) was added to each well, the plate was incubated for 60 min. Stop solution (50 μL) was added to each well, and the sample absorbance was measured at 505 nm by microplate reader (SpectraMax, CA, USA).

### Immunoblotting analysis

Mice were decapitated after behavioral tests, the hippocampus and prefrontal cortex were dissected and stored at −80 °C until analysis^1^. The hippocampal tissues were homogenized in RIPA lysis buffer containing protease and phosphatase inhibitors and centrifuged at 13 000 rpm for 30 min at 4 °C. Samples were separated using SDS-PAGE before transferring to nitrocellulose membranes. Blots were blocked with 4% BSA for 1 h, and incubated with the appropriate primary antibodies over night at 4 °C. The anti-Bcl-2 (sc-7382, 1:1000), anti-Bax (sc-7480, 1:500), anti-BDNF (sc-65514, 1:500), anti-β-actin (sc-8432, 1:2000), were purchased from StantaCruz Biotechnology, USA. The anti-IL-22 (ab181007, 1:1000), anti-IL-17 (ab79056, 1:1000), anti-PKG (ab97339, 1:1000) and anti-PKA (ab211265, 1:1000) anti-bodies were purchased from Abcam Inc, USA.).Then the membranes were incubated with the secondary antibodies (anti-mouse lgG-HRP (sc-2005, 1:5000) or anti-rabbit lgG-HRP (sc-2004, 1:5000)) for 60 min at room temperature. After rinsing with buffer, the immune complex was visualized by chemiluminescence using the ECL kit (Catalog number: 32134, Thermo Fisher Scientific, USA) according to the manufacturer’s instructions. The film signals were digitally scanned and then for subsequent analysis with software.

### Statistical analysis

All data were expressed as means ± standard error of the mean (SEM). All data were analyzed using ONE-WAY ANOVA except for the data from the acquisition training of the Morris water maze, which were analyzed using TWO-WAY ANOVA, followed by a post hoc Dunnett’s test. Difference with p < 0.05 were considered statistically significant.

### Data availability Statement

All the materials, data and associated protocols promptly available to readers without undue qualifications in material transfer agreements.

## Results

### BAY reversed Aβ-induced memory impairment in the Morris water maze task and step-down passive avoidance test

**Figure 2:**
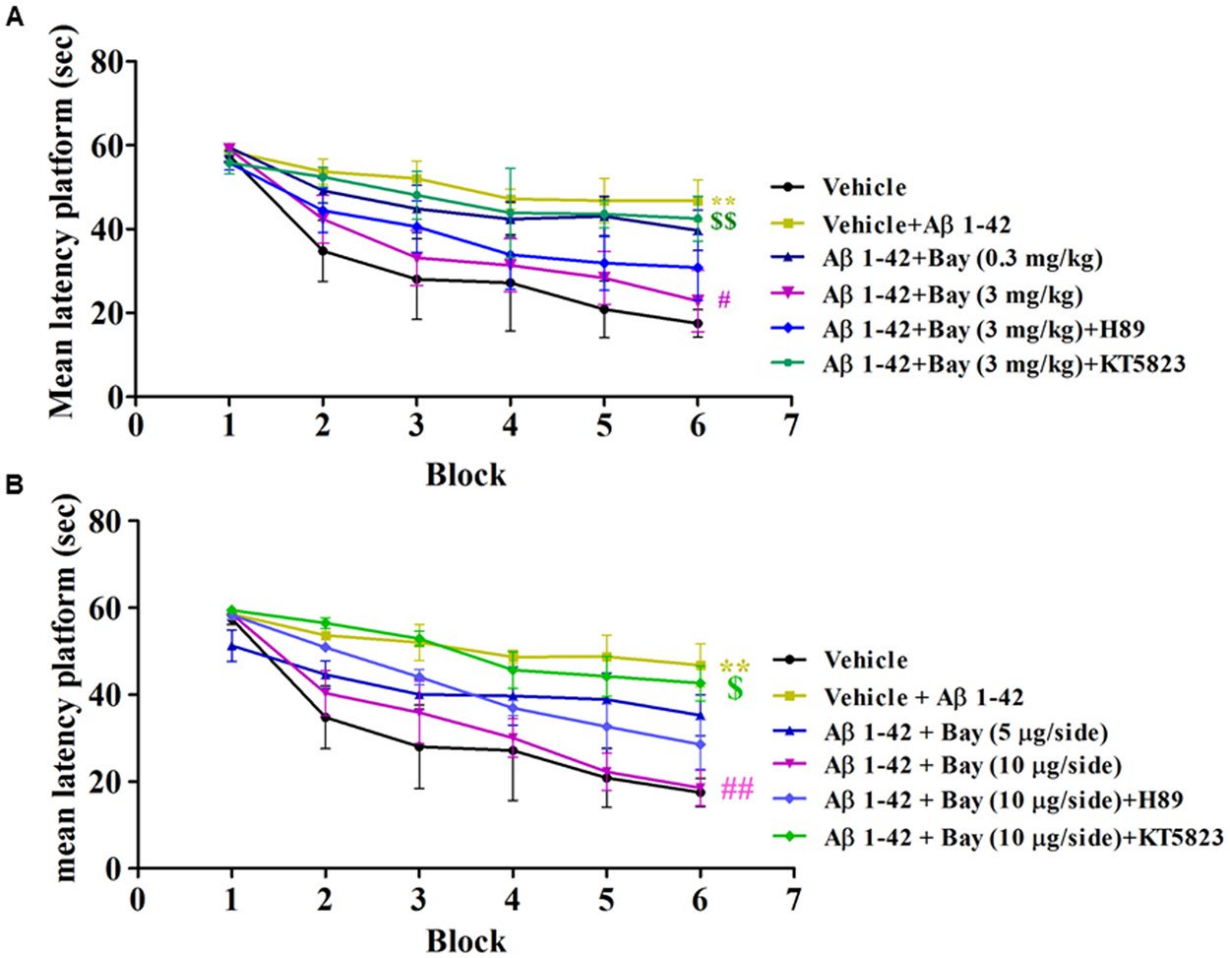
Learning curve in the water maze task after treatment with BAY 60-7550 (0.3 and 3 mg/kg, *i.p*. or 5, 10 μg, *i.c.v*.). Four days after microinfusion with Aβ 1-42 into cerebroventricle, mice were administrated with BAY for 14 days. H89 and KT5823 were pretreated 30 min before BAY administration every day. Behaviors were tested 24 h after last treatment (mean ± SEM, n = 8–10 for each condition). **p < 0.01 (5, 9.900), *vs* vehicle-treated control group. ^#^p < 0.05, *vs* vehicle-treated Aβ group. ^$$^p < 0.01, *vs* BAY (3 mg/kg)-treated Aβ group or BAY (10 μg)-treated Aβ group.

To determine whether BAY reversed Aβ 1-42-induced memory impairment, we observed memory performance in the Morris Water Maze test and step-down passive avoidance test in mice treated with Aβ 1-42 in the presence or absence of BAY via *i.p*. or *i.c.v*. Although all mice reliably learned to locate the platform throughout 6 blocks of acquisition training, the groups significantly differed in their latency to reach the platform during the 6 training blocks. The results showed that vehicle-treated Aβ (0.4 μg) group mice took longer time to reach the platform at block 6th compared to vehicle-treated control mice (F _(5, 150)_ = 15.29, p < 0.01; Fig. 2A), which were reversed by treatment with BAY at 3 mg/kg (*i.p*.) for 14 days, i.e. latencies to reach the platform for BAY (3 mg/kg via *i.p*.)-treated Aβ mice were significantly shorter than that of the vehicle-treated Aβ group in the 6th block (F _(5, 150)_ = 15.29 p < 0.05). Similar acquisition performance was observed from mice treated with BAY at 10 µg via *i.c.v*, which showed better performance in learning curve as shown in Fig. 2B (F _(5, 150)_ = 1.493, p < 0.01). While these effects of BAY (3 mg/kg by *i.p*. and 10 µg by *i.c.v*) on acquisition were weakened by pretreatment with PKG inhibitor KT5823 (p < 0.01; p < 0.05). The PKA inhibitor H89 partially blocked the effects of BAY on acquisition processes. KT5823 and H89 did not have effects on their own (data not shown).

**Figure 3:**
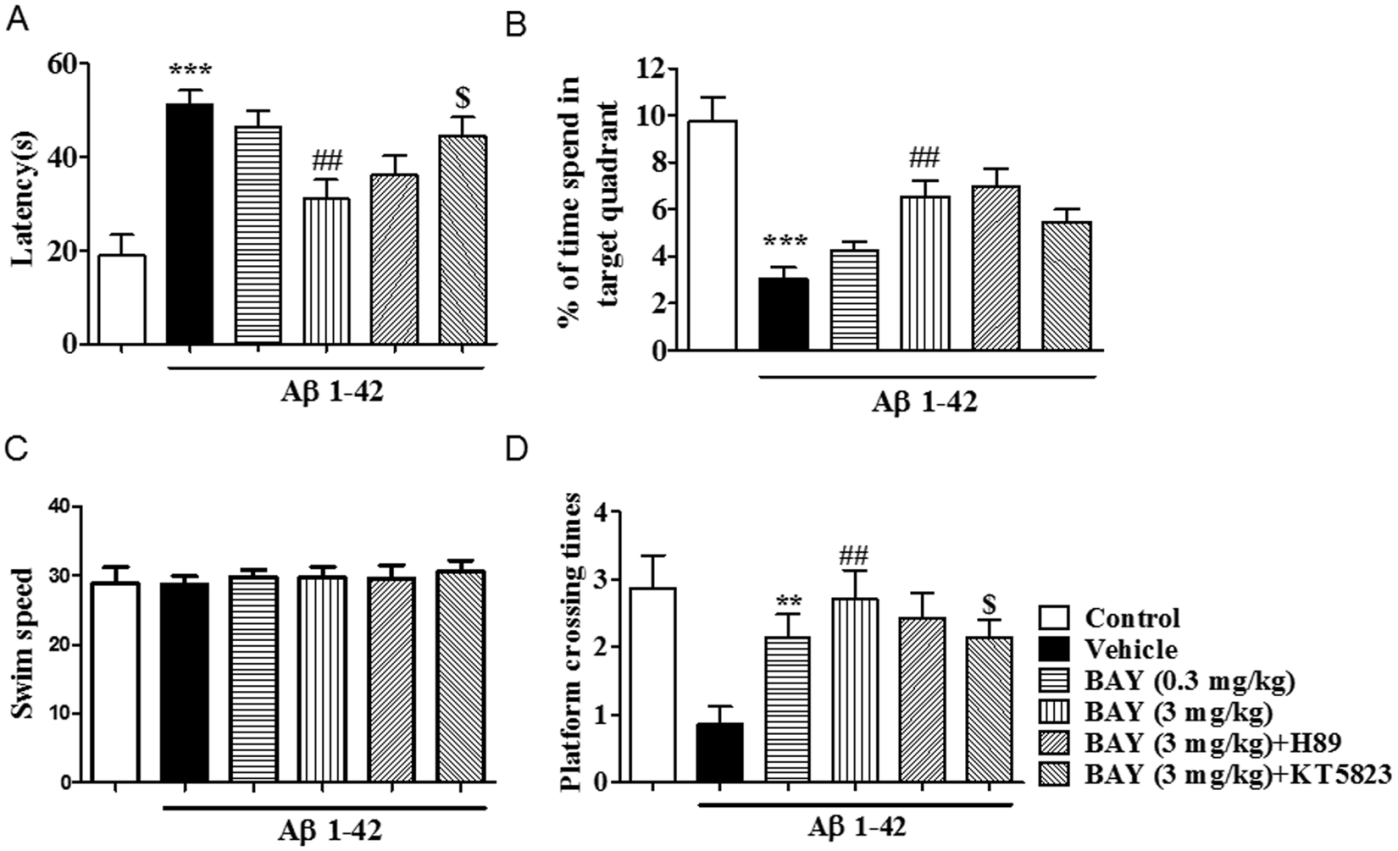
BAY 60-7550 (*i.p*.) reversed Aβ-induced memory impairment 24 h after training. During the 24 h probe trails of the water maze task, the latency to get the platform (**A**), percentage (%) of the time spend in the target quadrant (TQ) (**B**), and the platform crossing times (**D**) were tested after BAY treatment via *i.p*. for 14 days (mean ± SEM, n = 7–10). **p < 0.01, ***p < 0.001, *vs* vehicle-treated control group. ^##^p < 0.01, ^###^p < 0.001 *vs* vehicle-treated Aβ group. ^$^p < 0.05, ^$$^p < 0.05, *vs* BAY (3 mg/kg)-treated Aβ group. The swim speed was not altered among all the groups in the 24 h test (**C**).

**Figure 4:**
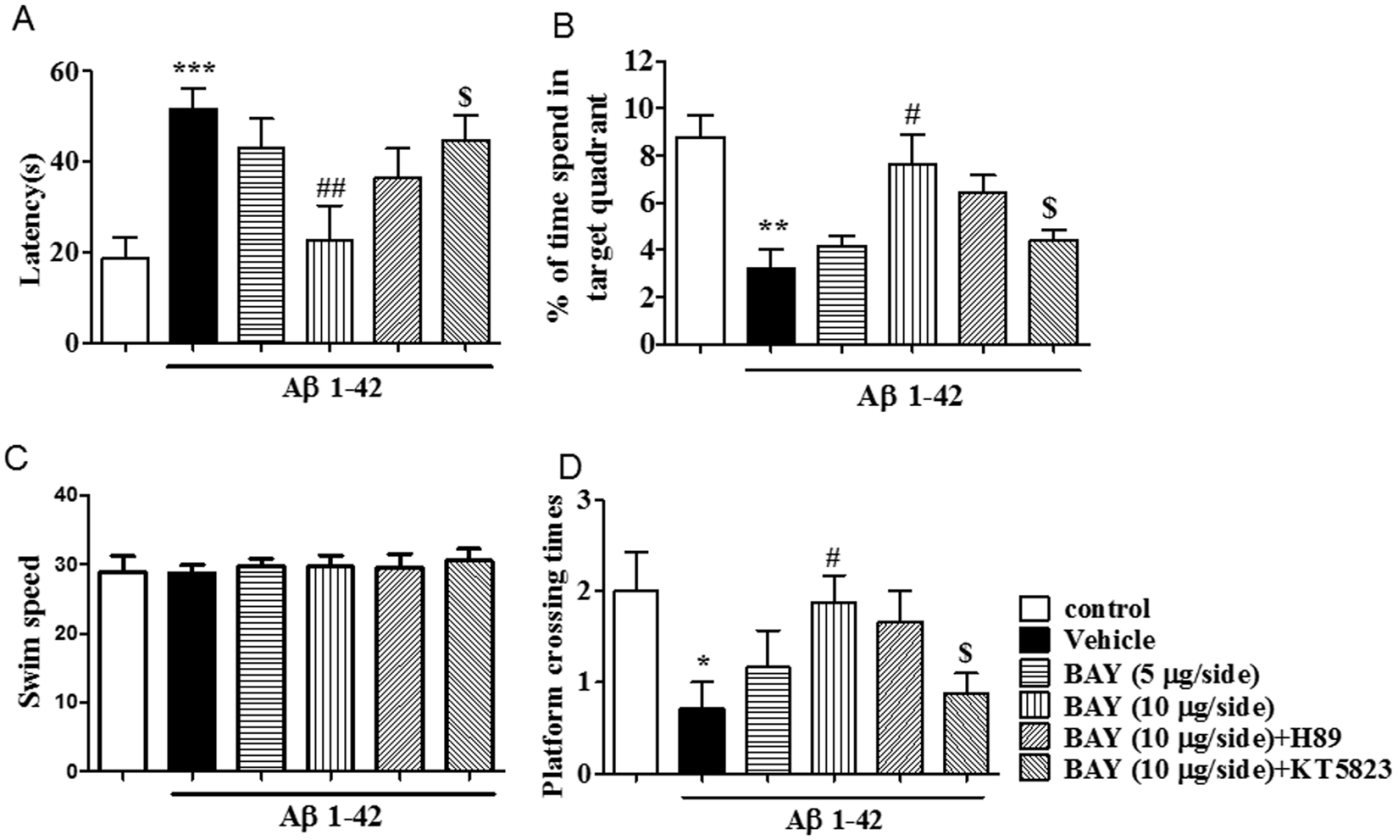
BAY 60-7550 (*i.c.v*.) reversed Aβ-induced memory impairment 24 h after training. During the 24 h probe trails of the water maze task, the latency to reach the platform (**A**), percentage (%) of the time spend in the target quadrant (TQ) (**B**), and the number of platform crossing (**D**) were tested after BAY treatment via *i.c.v* for 14 days (mean ± SEM, n = 10). *p < 0.05, **p < 0.01 and ***p < 0.001 *vs* vehicle-treated control group. ^#^p < 0.05, ^##^p < 0.01, *vs* vehicle-treated Aβ group. ^$^p < 0.05, *vs* BAY (10 μg/each)-treated Aβ group. The swim speed was not altered among all the groups in the 24 h test (**C**).

The 24 h memory retention for the platform location on the probe trial was tested after the training section. As shown in Figs 3 and 4, BAY (3 mg/kg by *i.p*. and 10 µg by *i.c.v*) ameliorated the detrimental effects of Aβ on platform latency (Fig. 3A, F _(5,44)_ = 9.90 p < 0.01; Fig. 4A, F _(5. 43)_ = 6.219, p < 0.01,(2.432, 49)), percentage of time spend in the target quadrant (TQ) (Fig. 3B, F _(5,37)_ = 11.82, p < 0.001; Fig. 4B, p < 0.001,) and number of platform crossings (Fig. 3D, F _(5, 37)_ = 3.473, p < 0.05; Fig. 4D, F _(5, 40)_ = 2.432 p < 0.01). Again, BAY’s effects were blocked by pretreatment with KT5823 (p < 0.05; p < 0.05), but, H89. The swim speed was not altered among all the groups in the 24 h test, which showed that surgery operation and compound treatment did not affect the animals’ abilities of vision and motor activity (Figs 3C and 4C).

**Figure 5:**
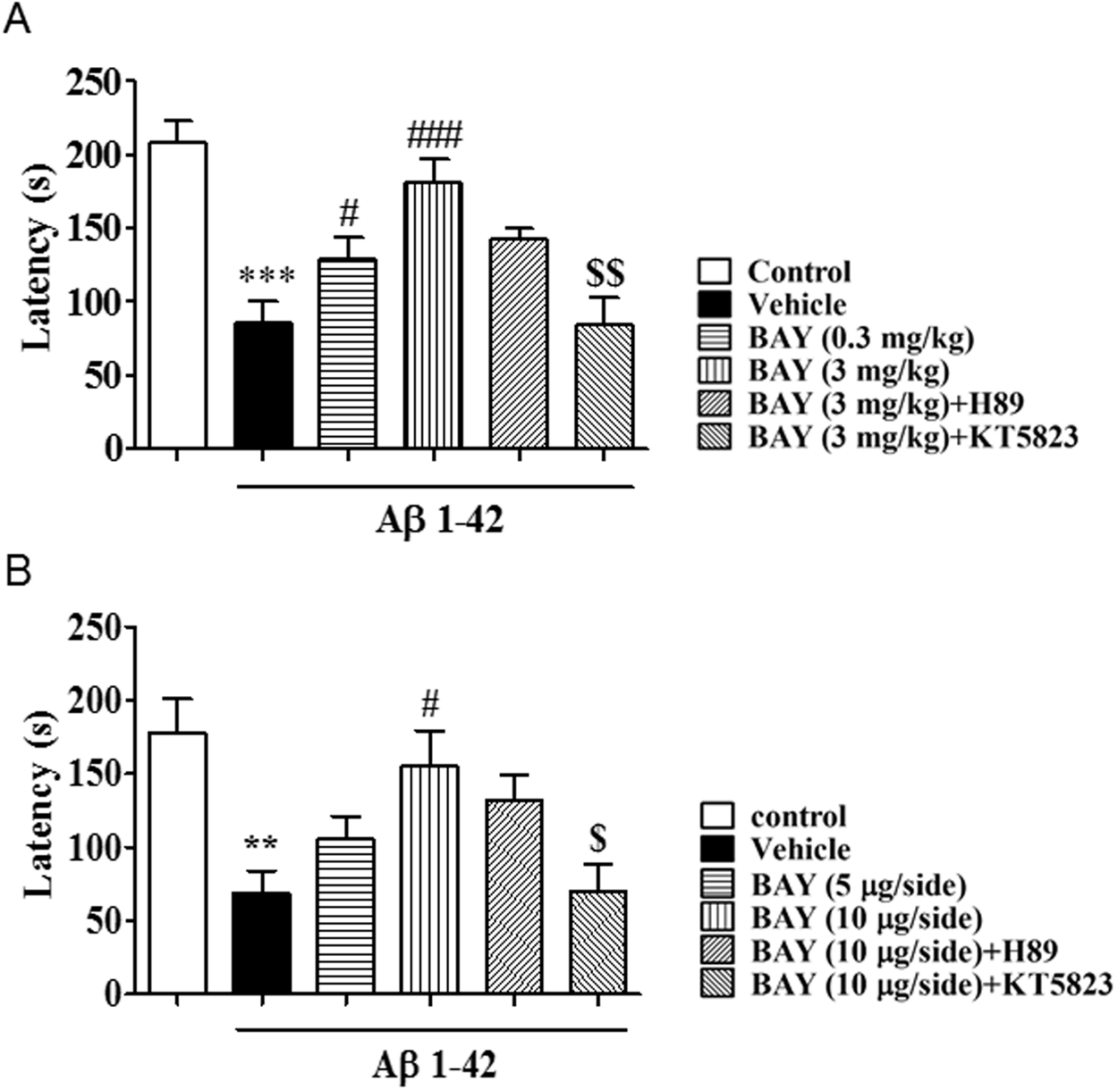
BAY 60-7550 in both of i.p. and i.c.v. administration reversed shorten of the latency to the first time in step-down passive avoidance test. (**A**) Retention in the step-down passive avoidance test in Aβ 1-42 treated mice. Aβ 1-42-induced decreases in 24-h retention were reversed by BAY (*i.p*. 3 mg/kg) treatment (mean ± SEM, n = 10). ***p < 0.001 *vs* vehicle-treated control group. ^#^p < 0.05, ^###^p < 0.001 *vs* Aβ 1-42-treated control group. ^$$^p < 0.01 *vs* BAY (*i.p*. 3 mg/kg) treated Aβ 1-42 group. (**B**) Retention in the step-down passive avoidance test in Aβ 1-42 treated mice. Aβ 1-42-induced decreases in 24-h retention were reversed by BAY (*i.c.v*. 10 μg/each) treatment (mean ± SEM, n = 10). **p < 0.001 *vs* vehicle-treated control group. ^#^p < 0.05 *vs* Aβ 1-42-treated control group. ^$^p < 0.05 *vs* BAY (*i.c.v*. 10 μg/each) treated Aβ 1-42 group.

As shown in Figs 5A and B, in the retention test performed 24 h after training, BAY (3 mg/kg by *i.p*. and 10 µg by *i.c.v*) showed significantly higher retention (F _(5, 30)_ = 11.08, p < 0.00; (F _(5, 32)_ = 5.405, p < 0.01). Again, BAY’s effects were blocked by pretreatment with KT5823 (p < 0.01; p < 0.05), but not H89.

### BAY reversed Aβ-induced increases in IL-22 and IL-17 levels in the cortex and hippocampus

**Figure 6:**
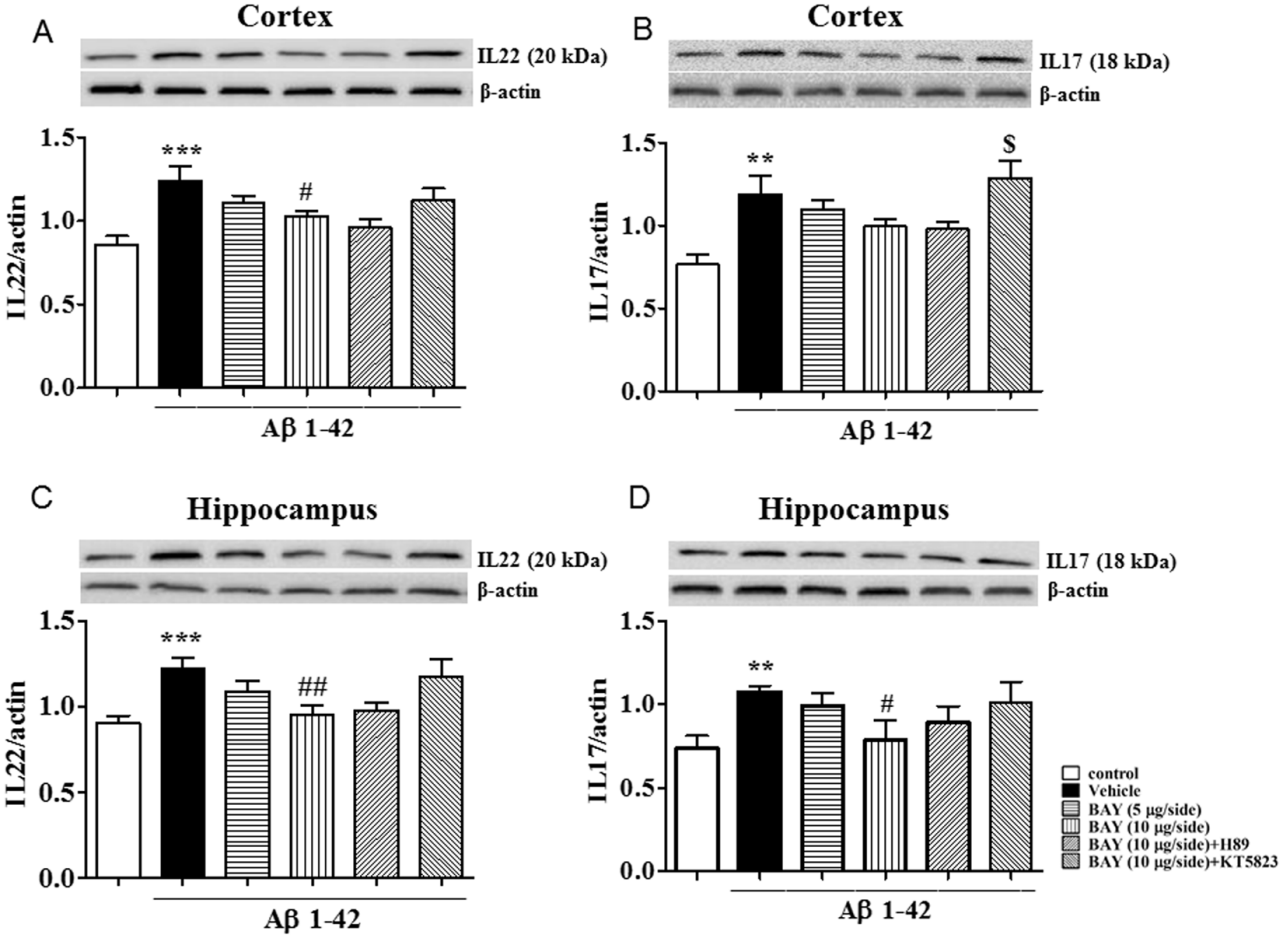
The effects of BAY 60-7550 on Aβ-induced changes in IL-17 and IL-22 expression. IL-22 and IL-17 expression after BAY (*i.c.v*.) treatment was tested in the cortex (**A**,**B**) and in the hippocampus (**C**,**D**) of mice (mean ± SEM, n = 10). **p < 0.01, ***p < 0.001, *vs* vehicle-treated control group. ^#^p < 0.05, ^##^p < 0.01, *vs* vehicle-treated Aβ group. ^$^p < 0.05, *vs* BAY (10 μg)-treated Aβ group.

To illustrate the effects of BAY on Aβ 1-42-induced neuroinflammatory responses in the cortex and hippocampus, we investigated pro-inflammatory cytokines expression, such as IL-22 and IL-17, in the presence or absence of BAY via *i.c.v*. As shown in Fig. 6, the IL-22 and IL-17 levels in the vehicle-treated Aβ 1-42 group were markedly increased in the cortex and hippocampus when compared with those of vehicle-treated control groups. The up-regulated IL-22 expression induced by Aβ 1-42 was significantly blocked by treatment with BAY at 10 μg via *i.c.v*. in both cortex and hippocampus (Fig. 6A and C, F _(5, 134)_ = 4.983, p < 0.05; F _(5, 53)_ = 3.929, p < 0.01). H89 and KT5823 did not obviously prevent the effects of BAY on IL22 expression. BAY induced a decreasing tendency of IL-17 expression in the cortex though the difference was not significant (Fig. 6B), which was reversed by pretreatment with KT5823. Increased IL-17 levels in the hippocampus induced by Aβ 1-42 were prevented by BAY at 10 μg via *i.c.v* (Fig. 6D, F _(5, 37)_ = 2.053, p < 0.05), which was partly blocked by pretreatment with KT-5823.

### BAY prevented Aβ-induced apoptotic response in the cortex and hippocampus

**Figure 7:**
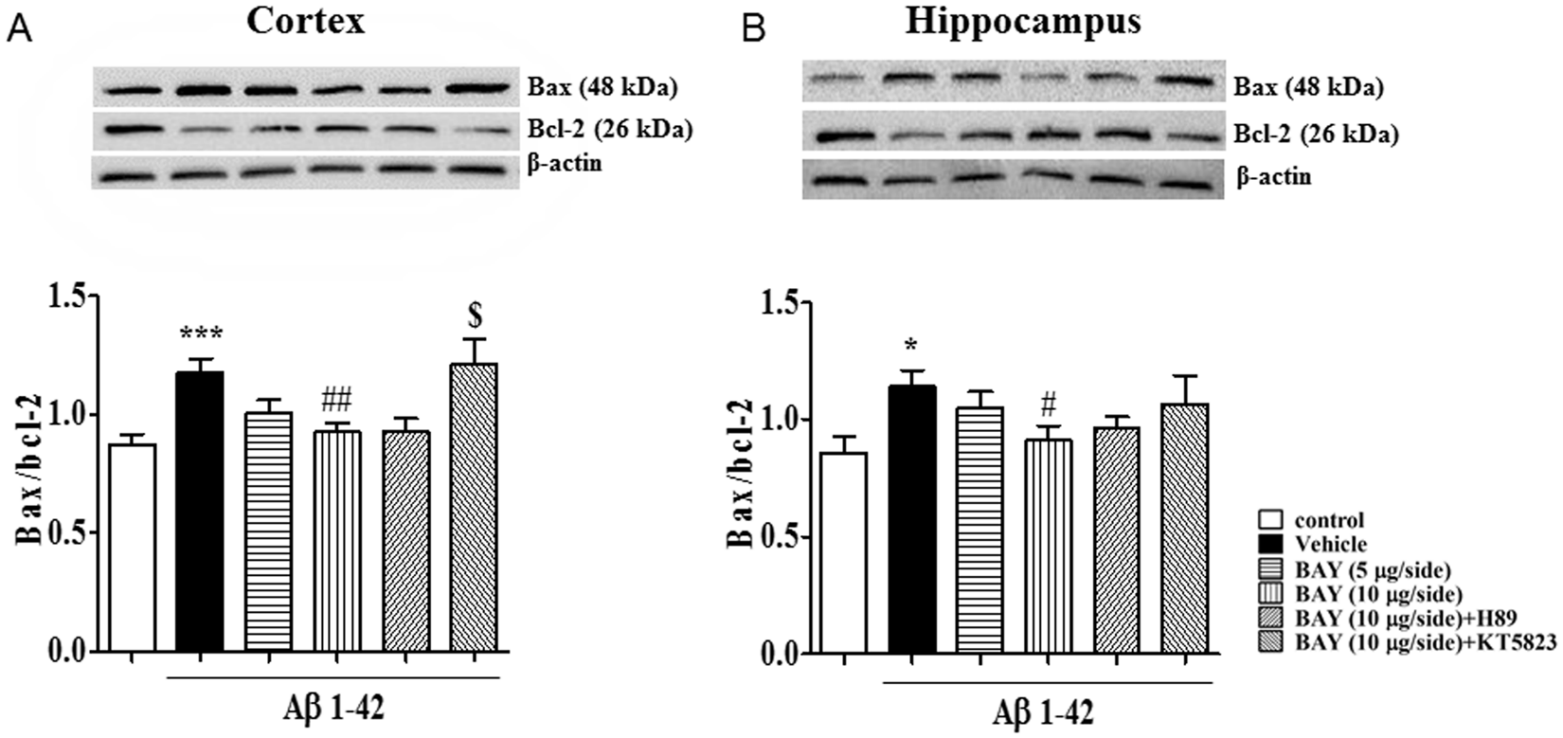
The effects of BAY 60-7550 on Aβ-induced increases in the ratio of Bax/Bcl-2. The ratio of Bax/Bcl-2 were determined after BAY (*i.c.v*) treatment in the cortex (**A**) and the hippocampus (**B**) of mice (mean ± SEM, n = 10). *p < 0.05, ***p < 0.001, *vs* vehicle-treated control group. ^#^p < 0.05, ^##^p < 0.01 *vs* vehicle-treated Aβ group. ^$^p < 0.05, *vs* BAY (10 μg)-treated Aβ group.

Aβ-induced cell apoptosis involves activation of series of pro-apoptotic markers and inhibition of anti-apoptotic proteins, such as Bax and Bcl-2. We determined the ratio of Bax to Bcl-2 to confirm whether anti-apoptotic events were involved in the effects of BAY on Aβ 1-42-induced neuro-inflammatory response. As shown in Fig. 7, the ratio of Bax and Bcl-2 was increased both in the cortex (F _(5, 118)_ = 4.710, p < 0.05, Fig. 7A) and hippocampus (F _(5, 36)_ = 1.949, p < 0.01, Fig. 7B) after intracerebroventricle microinfusion with Aβ 1-42. These effects were reversed by treatment with BAY (10 μg via *i.c.v*) in both of cortex (p < 0.05) and hippocampus (p < 0.01). KT5823 blocked the protective effects of BAY in cortex (Fig. 7A), while an increased tendency was also found in the hippocampus.

### BAY reversed Aβ-induced decreases in cGMP and/or cAMP levels in the cortex and hippocampus

**Figure 8:**
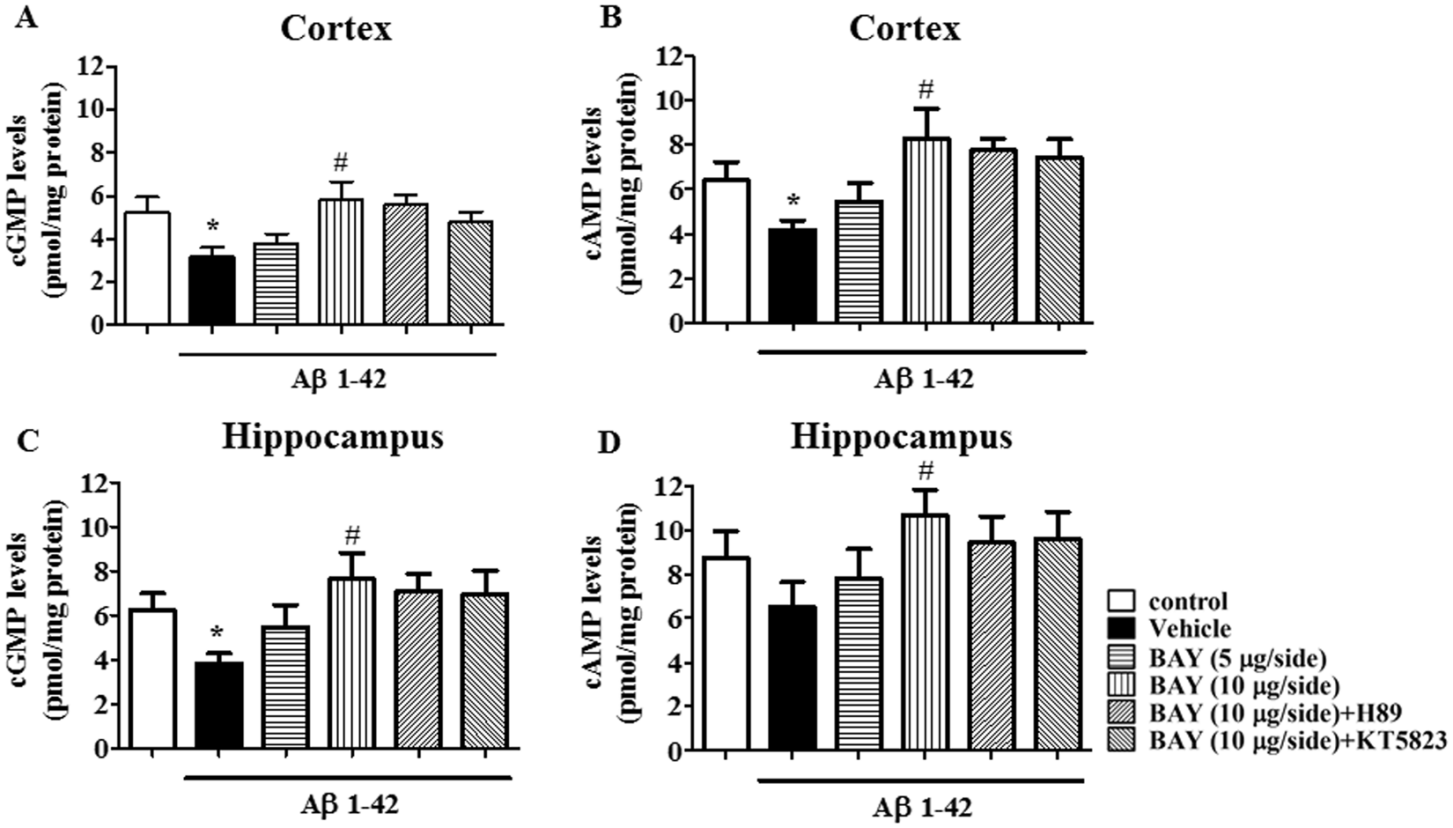
The effects of BAY 60-7550 on Aβ-induced decreases in cGMP and cAMP levels. The cGMP and cAMP levels after BAY (*i.c.v*.) treatment were determined in the cortex (**A**,**B**) and hippocampus (**C**,**D**) of mice (mean ± SEM, n = 10). *p < 0.05, *vs* vehicle-treated control group. ^#^p < 0.05, *vs* vehicle-treated Aβ group.

The changes in cGMP and cAMP levels in the cortex and hippocampus were observed after treatment with BAY in Aβ mice. The obvious decreases in cGMP levels in both cortex and the hippocampus were observed after Aβ 1-42 treatment (Fig. 8A and C, F_(5, 30)_ = 2.245, p < 0.05; F_(5, 30)_ = 5.304, p < 0.05). BAY treatment increased cGMP in both of hippocampus (p < 0.05) and cortex (p < 0.05). The cAMP level was also decreased in Aβ treated mice in the cortex (Fig. 8B), but not in the hippocampus though the decreased tendency was observed (Fig. 8D). The cAMP levels were also slightly increased in these brain regions after treatment with BAY (F _(5, 30)_ = 3.433, p < 0.05). KT5823 and H89 did not block the effects of BAY on cAMP or cGMP significantly.

### BAY blocked Aβ-induced decreases in PKA and/or PKG levels in the cortex and hippocampus

**Figure 9:**
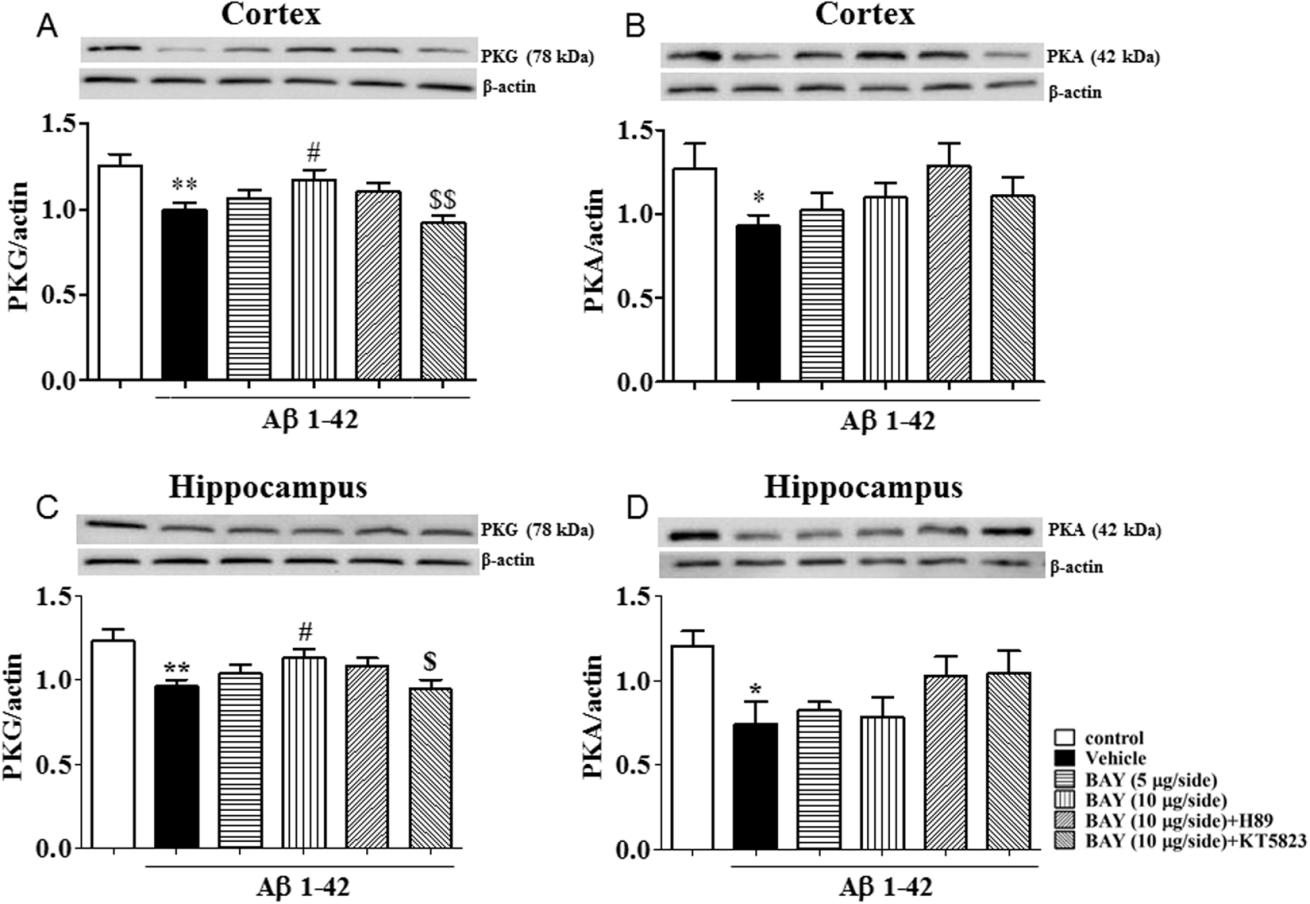
The effects of BAY 60-7550 on Aβ-induced decreases in PKA and PKG levels. PKG and PKA levels after BAY (*i.c.v*.) treatment were determined in the cortex (**A**,**B**) and hippocampus (**C**,**D**) of mice (mean ± SEM, n = 10). *p < 0.05, **p < 0.01, *vs* vehicle-treated control group. ^#^p < 0.05, ^##^p < 0.01, *vs* vehicle-treated Aβ group. ^$^p < 0.05, ^$$^p < 0.01, *vs* BAY (10 μg)-treated Aβ group.

To confirm whether Aβ 1-42 induced neurotoxicity involves PKA/PKG dependent pathway, we investigated PKA and PKG levels in the cortex and hippocampus. The significant decreases in PKG and PKA levels in both cortex and hippocampus were observed after treatment with Aβ (p < 0.01, p < 0.05). BAY at 10 μg via *i.c.v* significantly increased PKG levels in both cortex and hippocampus (F _(5, 115)_ = 5.170, p < 0.05 and F _(5, 94)_ = 3.655, p < 0.05; Fig. 9A and C). PKA levels were also slightly increased in the above two brain regions after treatment with BAY, but did not achieve the significant effect. KT5823 blocked the effects of BAY on PKG levels in both of the cortex and hippocampus.

### BAY reversed Aβ 1-42-induced decreases in BDNF expression in the cortex and hippocampus

**Figure 10:**
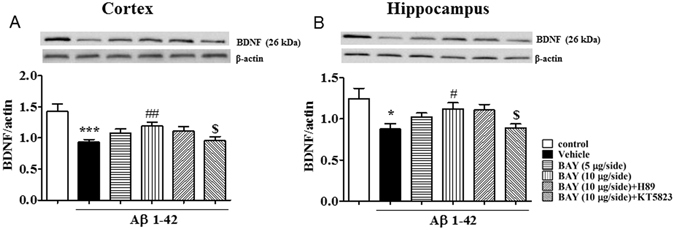
The effects of BAY 60-7550 on Aβ-induced decreases in BDNF expression. BDNF expression after BAY (*i.c.v*.) treatment were determined in the cortex (**A**) and hippocampus (**B**) of mice (mean ± SEM, n = 10). *p < 0.05, ***p < 0.001, *vs* vehicle-treated control group. ^#^p < 0.05, ^##^p < 0.01, *vs* vehicle-treated Aβ group. ^$^p < 0.05, *vs* BAY (10 μg)-treated Aβ group.

To determine whether the effects of BAY on memory performance is related to neuroprotective effects. We investigated BDNF expression in both the cortex and hippocampus in the presence of BAY in Aβ-treated mice. As shown in Fig. 10A and B, BDNF levels were decreased both in the cortex and hippocampus when mice were treated with Aβ (F _(5, 102)_ = 5.559, p < 0.001; F _(5, 83)_ = 2.836, p < 0.05). Such changes were blocked by BAY at 10 μg via *i.c.v* (p < 0.01; p < 0.05). KT5823 reversed the effects of BAY on BDNF expression in these two brain regions (p < 0.05 and p < 0.05), but not H89.

## Discussion

The present study demonstrates that PDE2 inhibition, by increasing cGMP signaling, is able to reverse Aβ 1-42-induced memory impairment in mice. The results demonstrated that PDE2 inhibitor BAY 60-7550, administered either *i.p*. or *i.c.v*, could ameliorate memory performance in the Morris water maze test in Aβ 1-42-induced mouse model of Alzheimer’s disease, which confirm the efficacy of the PDE2 inhibitor on learning and memory performance in the process of AD. The subsequent study suggested that BAY reversed Aβ-induced increases in IL-22, IL-17 and the ratio of Bax/Bcl-2, and decreased BDNF, cAMP and cGMP levels. The PKG inhibitor KT5823 attenuated the effects of BAY on both the behavior and the neurochemical changes, while the PKA inhibitor H89 did not show significant effects on reversing BAY’s action.

The present study suggested that Aβ 1-42 treatment led to extension of platform latency and fewer crossings over the platform. BAY-treated mice (both of *i.p*. and *i.c.v*) showed shorter latency to reach the platform and more crossings than Aβ-treated mice. Mice pretreatment with KT5823 before BAY partially turned back to the same behaviors as Aβ-treated mice did. Our results corroborated the findings that the memory-enhancing potential of BAY were significant in adult and aged animals previously reported^19,20^. It should be noted that in our study, the last dose of drugs were administrated 24 hours before the behavior tests started, and during the test days, the drugs were always administered at the end of the day, after behavioral tests completed, for the maintenance of drug effect. We used *i.p*. and *i.c.v* administration, two routes of drug administration to confirm the efficacy of the PDE2 inhibitor on memory processes. The results of comparison confirmed that both administration routes of BAY reversed Aβ-induced abnormality of behavior in the Morris water maze test. Bollen’s work suggested that both object memory and long-term potentiation (LTP) were improved after administration of two sub-efficacious doses of the dual substrate PDE2 inhibitor BAY (0.3 mg/kg) at the early and late consolidation phase^21^, which consists with our present findings. However, the bioavailability and the pharmacokinetic parameters of BAY are still not clear. Galindo-Tovar and co-workers investigated the effects of BAY on spontaneous beating of right atria in mice, which suggested that high dose of BAY increased basal sinoatrial beating^22^. Considering the side effect of high dosage administration of BAY, we performed the *i.c.v*. to directly deliver BAY into the cerebroventricle, making it infusion into brain regions rapidly. The results of behavior tests and immuno-blotting analysis ensured that *i.c.v*. administration of BAY (10 μg/side) significantly reversed Aβ-induced memory impairment, neuroinflammation and apoptosis of hippocampal neurons in mice. None of side effects were detected on BAY administrated mice (10 μg/side, *i.c.v*).

The essential role of PDE2 on process of aging and Alzheimer’s disease has been reported in previous studies^23,24^, which suggested that natural products such as resveratrol showed neuroprotective effect in Aβ-induced memory impairment. The effects are involved in its inhibition of PDE4 dependent signaling^1^. Indeed, PDE-cAMP/cGMP signaling is closely contributed to the regulation of neuroinflammation and cellular apoptosis as evidenced by changes in cytokines, pro-apoptotic and anti-apoptotic factors^25^. T cells are activated and infiltrate in the brain of AD patients, which alter a number of various subsets of T cells in the circulation in the process of AD^26^. The proinflammatory cytokines released by Th17 cells, such as interleukin (IL)-17 and IL-22, were elevated in the serum, cerebrospinal fluid and the hippocampus or cortex in AD occurrence and development^27^. These increased proinflammatory cytokines released by Th17 cells bind to their relevant receptors on neurons, resulting in neuronal apoptosis or death^28^. Moreover, evidence suggested that cAMP were also able to reduce the expression of pro-inflammatory cytokines, such as tumor necrosis factor α (TNF- α) and interleukin (IL)-17^29^. Porte and his co-workers have converged to conclude that basal CREB phosphorylation is lower in pyramidal cells of all sub-regions of the hippocampus of aged animals compared to young adult animals^30^. Additionally, there is limited activation of the cAMP-PKA-CREB cascade during learning in aged animals, which might also contribute to age-related cognitive deficits^23^.

The present study found that the up-regulated IL-22 and IL-17 expression in Aβ-treated mice was significantly reversed by PDE2 inhibitor BAY, while the effects of BAY were prevented by pretreatment with PKG inhibitor KT5823. These results indicate that PDE2-cGMP-dependent pathway may be involved in BAY’s effects on IL-22 or IL-17 stimulated neuronal inflammation.

It is interesting to know whether inhibition of PDE2 produces the anti-apoptotic effects in Aβ-treated mice. Several proteins are related to the progress of apoptosis, such as Bcl-2, Bax, caspase family members, p53 and p21^31,32^. Among them, the Bcl-2 family members are the most intriguing proteins in the regulation of apoptotic process. They can be divided into anti-apoptotic members, such as Bcl-2, Bcl-xl, and Bcl-w, and pro-apoptotic members, such as Bax and Bak^33,34^. It has been suggested that microinjection of Aβ into brain decreases Bcl-2 and increases Bax expression, leading to neuronal cell death in mice^35^. Our study found that Aβ 1-42 increased the ratio of Bax/Bcl-2 both in the hippocampus and cortex of mice, which were reversed by treatment with BAY. PKG inhibitor KT5823 reversed this effect of BAY, while PKA inhibitor H89 partially blocked the effect. These findings agreed with our previous studies that illustrated BAY regulated the abnormalities of pro- and anti-apoptotic components, such as Bax, Caspase 3 and Bcl-2, in the hippocampus and amygdala of stressed mice^36^. Indeed, PDEs function is linked to cell apoptosis in learning and memory processes^37^. As a member of nuclear envelop protein AKAP (A-kinase anchoring proteins) family, AKAP149 associated with mitochondria and plays an essential role in mitochondrial apoptosis. The present study implies that the regulation of neuronal apoptosis by PDE2 inhibition might be partly related to the difference in the structure and location of AKAP149-PKA/PKG-PDE complex. AKAP 149–PKA–PDE4A distribution and integrity have a key role in cellular survival^38^. Further studies is necessary to confirm whether BAY-induced change in the ratio of Bax/Bcl-2 is involved in PKG/PKA-PDEs complex that is localized in the nuclear domain of neuronal cells.

We found that the decrease in cGMP level in hippocampal tissue was more obvious than the cAMP level down regulation after Aβ 1-42 treatment. In accordance with the results of behavioral tests, KT5823 weakened the reversion of BAY on cGMP level in hippocampus but not in Cortex. As the down-stream of cAMP/cGMP-PKA/PKG-BDNF signal pathway, PKG regulation also showed as the same trends as that of cGMP levels. As a functional inhibitors, none of BAY60-7550, KT5823 or H89 is able to affect the target protein on the expressional level with a short term treatment, unless these small molecular compounds showed any side effect. In this study, the mice were treated with BAY or the other functional inhibitors in a long-term (14 days, 0.3, 3 mg/kg, i.p., or 5, 10 μg/side, i.c.v.). This kind of “chronic treatment” may cause a compensatory up-regulation in PKG or PKA level. S. Gambaryan *et al*. reported that oligopeptide DT-2, a novel PKG I inhibitor, led to a decrease in PKG expression in a dose dependent manner^39^. Meanwhile, L. Zhang *et al*. found that PKA selective inhibitor H89 up-regulated both p-PKA and total PKA expression in a time dependent manner^40^. In accordance with our results, these studies confirm the long term treatment of functional PKG or PKA inhibitors not only regulates the function, but also the expression of the target proteins.

Neurons in the hippocampus of aged animals respond to Aβ-induced toxicity by showing atrophy and a down-regulation of BDNF expression that are associated with learning and memory impairment^41^. Results from our study suggested that Aβ-induced a decreased BDNF level in the hippocampus and cortex of mice. It looks possible that the neuroprotective effects initiated by BAY might be mainly related to cGMP-signaling, because the PKG inhibitor KT5823 reversed BAY’s effects on BDNF expression. These findings in agreement with our previous study that suggested the selective PDE2 inhibitor BAY60-7550 prevents the breakdown of cAMP and/or cGMP and lead to increased BDNF expression^42^.

Taking together, the present study investigated that PDE2 inhibitor BAY ameliorates Aβ 1-42 induced learning and memory impairment. We also found that BAY exerts its neuroprotective effects by regulating the cytokines and apoptotic process-related biomarkers, such as IL-22, IL-17, and the ratio of Bax and Bcl-2, and BDNF expression. It is possible that inhibition of PDE2 by BAY 60-7550 attenuates the inflammatory and apoptotic responses via up-regulation of cAMP/cGMP-PKA/PKG-BDNF pathway in the brain, which in turn ameliorates cognitive behaviors.
